# Prevalence and causes of visual impairment in a Brazilian population: The Botucatu Eye Study

**DOI:** 10.1186/1471-2415-9-8

**Published:** 2009-08-19

**Authors:** Silvana Artioli Schellini, Shane R Durkin, Erika Hoyama, Flavio Hirai, Ricardo Cordeiro, Robert J Casson, Dinesh Selva, Carlos Roberto Padovani

**Affiliations:** 1 Faculdade de Medicina de Botucatu – UNESP – São Paulo State, Brazil; 2 South Australian Institute of Ophthalmology, Adelaide, Australia; 3 Discipline of Ophthalmology and Visual Sciences, The University of Adelaide, South Australia, Australia

## Abstract

**Background:**

This paper reports population-based data on the prevalence and causes of visual impairment among children and adults in Botucatu, Brazil.

**Methods:**

A population-based cross-sectional study was conducted involving a random start point and then systematic sampling of an urban Brazilian population in the city of Botucatu. There were approximately 3 300 individuals aged 1 to 91 years who were eligible to participate in the study. Of this sample, 2485 (75.3%) underwent ophthalmic examination. The ophthalmic examination included uncorrected (presenting) and best corrected distance visual acuity using standardized protocols. The primary cause of decreased visual acuity was identified for all patients with visual impairment.

**Results:**

Presenting low vision and presenting blindness were found in 5.2% (95% CI: 4.3–6.1) and 2.2% (95% CI: 1.6–2.8) of the population, respectively. Unilateral presenting low vision and unilateral presenting blindness were found in 8.3% (95% CI: 7.2–9.5) and 3.7% (95% CI: 2.9–4.4) of the population respectively. Best corrected low vision was found in 1.3% of the population (95% CI: 0.9–1.7) and best corrected blindness was discovered in 0.4% of people (95% CI: 0.2–0.7). The main cause of presenting low vision was refractive error (72.3%) and cataract was the most prevalent cause of blindness (50%).

**Conclusion:**

The main causes of low vision and blindness in this Brazilian city were uncorrected refractive errors, cataract, and retinal diseases. Programs to further reduce the burden of visual impairment need to be targeted toward the correction of refractive error and surgery for cataracts.

## Background

In 2002, the World Health Organization (WHO) estimated that there were in excess of 161 million people worldwide with visual impairment (corrected VA < 6/18 in the better eye), including 37 million with blindness (corrected VA < 3/60 in the better eye) [1,2]. If this definition is expanded to include uncorrected refractive error then it is estimated that 259 million people are visually impaired [3]. The burden of blindness is particularly severe in South-East Asia and India; however in countries such as Brazil, the prevalence of blindness is estimated to be 1.3% of those over the age of 50 years [2]. This represents not only a significant burden to those who are affected, but it also represents a large social and economic cost to the State [4].

There are a paucity of population-based data relating to the level of visual impairment and its causes within Brazil. It is therefore imperative that accurate ophthalmic epidemiological data from this region is collected in order to optimize the allocation of limited resources. There is some evidence from retrospective data that the leading causes of visual impairment in Brazil are uncorrected refractive error and cataract, closely followed by age-related macular degeneration and glaucoma [5].

This paper presents population-based information on the prevalence and causes of visual impairment in Botucatu, Brazil. Unlike many other studies into the prevalence of visual impairment, this study also included children. These data will be of assistance in planning for the delivery of eye health care and the implementation of programs to further reduce the burden of blinding disease by the year 2020.

## Methods

### Sampling procedure

The Botucatu Eye Study (BES) was a population-based, cross-sectional ophthalmic survey of people living in households in the city of Botucatu, Brazil. Botucatu is a municipality in the state of São Paulo in Brazil, located 225 km from São Paulo, the state capital and 898 km from Brasília. It lies at 22°53'09"S, 48°26'42"W, has a temperate climate, and covers a total area of 1486.4 km^2^. The population in 2001 was 108,306 with 70% of people having less than 10 years of formal education and manufacturing being the most common mode of employment. The principal aims of this project were to estimate the prevalence and causes of visual impairment and the prevalence and risk factors of ocular disorders among all age groups of people in this region.

The population census data (Instituto Brasileiro de Geografia e Estatsitica (IBGE), 2001) for the Botucatu census sectors was used as the sampling frame. The eligible population consisted of permanent, non-institutionalized residents of Botucatu over the age of 1 year.

A sample size calculation was then made based on the population size of Botucatu and the assumption that the prevalence of visual impairment (as defined below) in Botucatu was thought to be approximately 2% [2]. In order to determine this level of visual impairment with a precision of 0.5% and level of significance of 5%, it was calculated that 2931 participants would be required for the study. It was determined that the average number of household residents was 3.3. Sampling 1 000 households would therefore yield and estimated eligible population of 3 300.

The IBGE divided the city into census sectors and households were identified within each block and enumerated in sequence to cover the entire urban area. The households were to be examined were selected using a random start point with a household cluster sampling technique. Households were identified systematically according to local census data: the first house was selected randomly; the next house was the seventh house on the even-numbered side of the street and so on, successively until the pre-determined number of households in each census sector had been reached. The number of households in each sector was determined based on the proportion of people in that sector compared to the total Botacatu population.

Each of the selected households received a letter of invitation to participate in the study, which was followed by a field worker visit. Those who agreed to participate were contacted by telephone to schedule an appointment at the Botucatu School of Medicine University Hospital, where all of the examinations were conducted. All persons of the household were eligible to participate in the study if it was their usual place of residence (by self-report) and they had attained the age of 1 year. If there was no answer when the examiners contacted the household or if people refused to participate in the research, the first house to the right was selected. If the next household refused to participate, the first house to the left of the initial house was selected, and so on, successively. After inviting 1,000 households to participate in the survey (approximately 3 300 people), 2 485 individuals or 75.3% of the potential sample participated. The demographics of the examined population were compared to those of the entire Botacatu population at the time of the census.

### Data Collection

The study was carried out over a 4 month period by a single survey team consisting of 5 members, including 4 ophthalmologists. All study personnel underwent training and all procedures were standardized prior to commencement. Specific observations were performed by 1–2 members of the team in order to minimize interobserver variability. A medical and ophthalmic history was obtained from each patient in Portuguese by qualified health care workers. This included the collection of demographic, ocular and general health details.

Each participant then received a comprehensive vision and eye examination where uncorrected visual acuity (VA) was measured for the right eye followed by the left with a consistently illuminated illiterate E Snellen chart at 5 meters. The VA was then retested with the patients existing refraction. If the corrected VA was less than 20/20 an objective refraction using a streak retinoscope, trial frames and lenses was performed by the same two ophthalmologists (SAS, EH). This was subjectively refined using the Jackson cross-cylinder technique and the best corrected visual acuity (BCVA) recorded using the result of this refraction. If the subject was unable to read the largest letter at 5 m with the subjective refraction, testing was repeated at 1 m. If they were unable to read the largest letter at 1 m then the VA was recorded as count fingers (CF), hand movements (HM), light perception (LP) or no light perception (NLP). The spherical equivalent (SE) was calculated as the spherical error plus half the cylindrical error. The intraocular pressure (IOP) was measured using a noncontact pneumotonometer (CT-60 computerised tonometer, Topcon, Tokyo, Japan) and the mean of 3 measurements was recorded. If the IOP was higher than 25 mmHg then the measurements were repeated. Where possible, children under the age of five years had their visual acuity measured with the same chart; pre-verbal children were able to indicate the direction of the optotype. In those too young to do this a pupil examination, dilated fundus examination and cycloplegic refraction was able to exclude all but cortical causes of visual impairment or blindness. The intraocular pressure was not measured in children under 5 years unless clinically indicated.

Slit lamp biomicroscopy (BQ-900, Haag Streit, Bern, Switzerland) was performed, followed by dilatation with cyclopentolate 1%, 1 drop every 5 minutes (total of 3 drops for each eye) for those under the age of 13 years, cyclopentolate 1% 1 drop in each eye for participants aged 14–39 years, and tropicamide 1% and phenylephrine 10% for dilatation of those participants 40 years and over. The subjects then underwent fundus examination utilizing the slit lamp with a 90D Volk lens and then using a Schepens indirect binocular ophthalmoscope with a 20D Volk lens.

If best corrected VA was < 20/40 a determination as to the major cause of visual impairment in the best eye was made by the examining ophthalmologist at the end of the examination. Cataract was determined if the level of lens opacity correlated with the visual acuity, glaucoma was diagnosed based on intraocular pressure and optic nerve head appearance, and refractive error was the cause if the vision corrected to better than 20/30 with spectacles. If the etiology of visual impairment was not apparent the subject was scheduled for further investigations at the Botucatu School of Medicine University Hospital, after which a consensus diagnosis for the major cause of visual impairment was reached by two ophthalmologists.

### Definitions of low vision and blindness

Visual impairment was defined according to the following World Health Organization (WHO) categories [6]. *Presenting blindness *was defined as unaided VA (or with spectacles if worn) < 20/400 (3/60) in the better eye; and *WHO blindness *was defined as best corrected VA < 20/400 (3/60) in the better eye. *Presenting low vision *was defined as VA < 20/60 (6/18) but ≥ 3/60 (20/400) in the better eye, unaided (or with spectacles if worn). *WHO low vision *was defined as best corrected VA < 20/60 (6/18) but ≥ 20/400 (3/60) in the better eye.*Presenting visual impairment *was defined as the combined set of presenting low vision and presenting blindness, and *WHO visual impairment *was defined as the combined set of WHO low vision and WHO blindness. Field defects were not taken into consideration. Vision after subjective refraction was considered best-corrected vision for the purposes of the study.

### Ethics

The BES was approved by the Institutional Review Board and Research Ethics Committee of the Botucatu School of Medicine. Informed consent was obtained from participants prior to the commencement of the study. Where children were under the age of 16 years, their parents gave consent on their behalf. The study was then conducted in accordance with the tenets of the Declaration of Helsinki. The treatment of conditions found during the course of the study was offered by referral to the participants own ophthalmologist. If the individual did not have an ophthalmologist, they were referred to the Botucatu Medical School's Ophthalmology service.

### Statistical analysis

The prevalence of low vision and blindness were calculated based on the sampling design, which was approximated as a one-stage cluster design where each household was considered to be the primary sampling unit. Households were randomly selected and thus point prevalences were unbiased. Age was categorized into decades from 1–9 years through to those people 70 years and over. Ethnicity was categorized by self-identification into people of European, African-Brazilian, East Asian or other racial (including multiracial) descent. Occupation was categorized into professional, tradesperson, service worker (for example; secretary), manual worker (for example; construction worker), retirees, and students. Medical history was categorized into healthy, other, diabetes, hypertension and diabetes and hypertension. Univariate and then multivariate analyses were performed to determine whether age, gender, ethnicity, occupation or medical history was significantly related to blindness. Logistic regression models were constructed to investigate the combined predictors of refractive error in a multivariate fashion. Covariates were added in a stepwise pattern and retained if the R^2 ^increased, and beta coefficients were calculated for each predictor. Odds ratios (ORs) and 95% confidence intervals (CIs) for the predictors were calculated. All *P *values were 2-sided and were considered statistically significant when the values were <0.05. The statistical software packages used were SPSS version 13.0 (SPSS Inc., USA) and SAS Version 9.1 (SAS Institute Inc., Cary, NC, USA).

## Results

There was an assumed sample of 3 300 subjects eligible to participate, of whom 2485 (75.3%; 95% CI 73.8–76.7) had ophthalmic examinations. Table 1 compares the demographics of those participants examined to those of the greater Botucatu population and the municipality of Sao Paulo. The main reason for failure to participate was involvement in occupation-related activities. A major cause of visual impairment could be determined in all but one subject who required an electroretinogram in order to determine the disease process. The mean age was 38 years (range 1 to 91) and women comprised 57.5% (95% CI 55.6–59.4) of the study population. Approximately half of the population (49.9%, 95% CI 47.5–51.4) was above the age of 40 years (Table 1).

**Table 1:** The age group and gender of participants examined compared to the age and gender distribution of the inhabitants of the municipalities of Botucatu and Sao Paulo.

Age Group (years)	Examined n = 2485 (%)	Botucatu^# ^ n = 108 306 (%)	Sao Paulo^# ^ n = 10 435 546 (%)
1–9	213 (8.6)	17 629 (16.3)	1 708 230 (16.4)
10–19	372 (14.9)	20 459 (18.9)	1 878 255 (17.9)
20–29	355 (14.3)	18 695 (17.2)	1 969 018 (18.9)
30–39	317 (12.8)	16 124 (14.9)	1 699 367 (16.3)
40–49	457 (18.4)	13 966 (12.9)	1 352 029 (13.0)
50–59	348 (14.0)	9 292 (8.6)	855 640 (8.2)
60–69	216 (8.7)	6 442 (5.9)	540 687 (5.2)
70 and over	207 (8.3)	5 699 (5.3)	432 320 (4.1)
Gender		n = 90 677*	n = 8 727 317*
Male	1056 (42.5)	43 851 (48.4)	4 106 513 (47.1)
Female	1429 (57.5)	46 826 (51.6)	4 620 804 (52.9)

# Data obtained from the Instituto Brasileiro de Geografia e Estatsitica, 2001. * The municipal data on gender was only available for inhabitants aged 10 years and over.

Participants of European descent comprised 80.6% (95% CI 79.0–82.1) of the sample, followed by people of other races (14.1%; 95% CI 11.9–15.5), African-Brazilian (4.9%; 95% CI 4.1–5.8) and East Asian (0.4%; 95% CI 0.2–0.7) origin. Manual workers were the most common occupation group (25.6%; 95% CI 23.9–27.4), followed by students (22.5%; 95% CI 20.9–24.2), service workers (18.5%; 95% CI 17.0–20.1), professionals (13%; 95% CI 11.7–14.4), retirees (11.1%; 95% CI 9.9–12.4), and trades people (9.3%; 95% CI 8.2–10.5). The majority of the participants had no medical co-morbidities, (81.95%; 95% CI 80.4–83.4) the remainder of the sample suffered from 1 or more disease(s).

Blindness according to the WHO criteria was identified in 10 subjects and low vision in 32 subjects. The sampling design adjusted prevalence of WHO defined blindness was therefore, 0.4% (95% CI: 0.2–0.7) and low vision was 1.3% (95% CI: 0.9–1.7). The prevalence of presenting low vision was 5.2% (4.3–6.1) and the prevalence of presenting blindness was 2.2% (1.6–2.8). The age-specific prevalence of presenting and WHO-defined low vision, blindness, and visual impairment is presented in Additional File 1.

The causes for low vision, blindness and visual impairment are presented in Additional File 2. Refractive error (72.3%; 95% CI 63.8–79.8) and cataract (15.5% 95% CI 9.7–2.8) were the leading causes of presenting low vision. This was also true for presenting blindness where refractive error accounted for 66.7% (95% CI 52.5–78.9) and cataract led to 18.5% (9.3–31.4) of presenting blindness. The majority of WHO-defined low vision was ascribed to cataract (50.0%; 95% CI 31.9–68.1), and this was also true for WHO-defined blindness (50.0%; 95% CI 18.7–81.3).

The age-specific causes of presenting visual impairment were also calculated. These data are demonstrated in table 2. It can be seen that the most common cause of presenting visual impairment among those aged under 20 years was refractive error (97.3%), for those aged 21–50 years it was also refractive error (90.2%), and for those aged over 50 years it was cataract (50.0%).

**Table 2:** Age-specific cause of presenting visual impairment.

**Eye Pathology**	**Under 20 years** **Prevalence (%)**	**21–50 years** **Prevalence (%)**	**Over 50 years** **Prevalence (%)**
Cataract	0 (0.0)	3 (3.2)	27 (50.0)

Refractive Error	37 (97.3)	83 (90.2)	10 (18.5)

ARMD	0 (0.0)	0 (0.0)	9 (16.7)

Glaucoma	0 (0.0)	0 (0.0)	4 (7.4)

Maculopathy	0 (0.0)	3 (3.3)	2 (3.7)

Retinopathy	1 (2.7)	2 (2.2)	2 (3.7)

Optic Neuropathy	0 (0.0)	1 (1.1)	0 (0.0)

**Total**	38 (100)	92 (100)	54 (100)

ARMD = Age-related macula degeneration

Presenting visual impairment is the combination of those people with VA < 20/60 but ≥ 20/400 in the better eye, unaided (or with spectacles if worn) and unaided VA (or with spectacles if worn) < 20/400 in the better eye.

Both the univariate and multivariate analyses showed an increase in the prevalence of presenting visual impairment with age. (Table 3) For each year of increasing age there is a 3.0% increase in the odds of blindness (P = 0.002). In the multivariate analysis neither ethnicity nor general health had an effect on the odds of presenting visual impairment. Females had increased odds of presenting visual impairment (OR 1.5, p = 0.02) as did people who were retired (OR 2.2, p = 0.02).

**Table 3:** Association of presenting visual impairment with gender, age, ethnicity, occupation and general health.

**Variable**	**Univariate** **Odds Ratio (95% CI)**	**P Value**	**Multivariate** **Odds Ratio (95% CI)**	**P Value**
**GENDER**				
Male	1.0			
Female	1.4 (0.99–1.8)	0.06	1.5 (1.1–2.3)	0.02

**AGE (Years)**				
**Linear**				
1	1.0		1.0	
Each year after 1 year	1.03 (1.02–1.033)	<0.001	1.03 (1.01–1.05)	0.002
**Categorical**				
0–20	1.0		1.0	
21–50	2.8 (1.7–4.7)	<0.001	1.7 (0.60–4.5)	0.32
Over 50	4.1 (2.4–6.9)	<0.001	1.8 (0.62–5.4)	0.27

**ETHNICITY**				
European	1.0		1.0	
Brazilian-African	1.4 (0.75–2.8)	0.26	1.5 (0.74–2.8)	0.28
East Asian	1.4 (0.17–10.7)	0.78	1.2 (0.14–10.2)	0.87
Other	0.82 (0.50–1.4)	0.44	0.85 (0.49–1.5)	0.55

**OCCUPATION**				
Professional	1.0		1.0	
Tradesperson	0.78 (0.38–1.6)	0.49	0.75 (0.34–1.6)	0.47
Service worker	0.81 (0.46–1.4)	0.48	0.77 (0.41–1.4)	0.41
Manual worker	1.1 (0.68–1.9)	0.62	1.0 (0.58–1.8)	0.98
Retired	2.2 (1.3–3.8)	0.005	2.2 (1.1–4.2)	0.02
Student	0.41 (0.21–0.78)	0.006	0.53 (0.18–1.6)	0.25

**GENERAL HEALTH**				
1 or more health problems	1.0		1.0	
Healthy	0.59 (0.42–0.85)	0.004	0.94 (0.62–1.4)	0.79

Presenting visual impairment is the combination of those people with VA < 20/60 but ≥ 20/400 in the better eye, unaided (or with spectacles if worn) and unaided VA (or with spectacles if worn) < 20/400 in the better eye. CI = confidence interval.

There were no children with presenting low vision; however there was one child with presenting blindness due to high myopia that was correctable with spectacles. Again there were no children with WHO-defined blindness, but there was one child with WHO-defined low vision due to albinism.

## Discussion

Although there has been a considerable reduction in the infective causes of blindness, the global burden of blindness has not significanlty altered for over a decade [7,8]. The prevalence of blindness has been perpetuated by a number of factors including: increasing life expectancy in developing countries and the consequent increase in cataract and glaucoma; and the maldistribution of ophthalmic health care. The prevalence of blindness in Brazil is not immune to these factors and retrospective data has previously demonstrated the leading causes of visual impairment to be refractive error and cataract, followed by age-related macular degeneration and glaucoma [5]. These results were determined from the "Cataract Free Zone" project that was simultaneously commenced in Brazil and Peru in 1986 [9]. This paper highlights more recent population-based data on visual impairment in Botucatu, Brazil.

The level of presenting visual impairment of 7.4% in this paper is reduced compared to that found in regions across Brazil between 1986 and 1995 [5]. It may be that the continued efforts of the programs from which these data were drawn have been pivotal in the further reduction of visual impairment. The cause profile of this visual impairment has; however, not altered to a great extent.

In this study, refractive errors and cataract contributed to over 85% of presenting visual impairment and over 60% of WHO-defined visual impairment. These findings echo those previously demonstrated by Arieta et *al *[5] and are also reflected in data on visual impairment from around the world [2], not only in developing nations, but also in developed countries [10]. The correction of refractive error alone would lead to a significant decrease in the rates of both low vision and blindness. The uncorrected VA was better than 20/30 in just over 50% of the sample. The proportion of individuals with VA better than 20/30 increased to over 70% with correction. It is important to note the high level of presenting visual impairment among younger people aged 20–29 years (7.9%) and 30–39 years (10.4%). This seems disproportionately high but when the best corrected data are examined this falls to 1.4% and 0.6% respectively. This highlights the significant burden of visual impairment due to refractive error that is present is this younger, productive age group.

Aside from refractive errors and cataract, other causes of presenting visual impairment were: age-related macular degeneration (4.9%); glaucoma (2.2%); macular toxoplasmosis (2.7%); other retinopathy, which included diabetic retinopathy, albinism, retinal detachment, macula hole, retinitis pigmentosa, central retinal vein occlusion and central serous retinopathy (2.7%), and; optic neuropathy (0.5%). WHO-defined visual impairment demonstrated a similar profile; however, the relative frequencies of age-related macular degeneration (7.1%); glaucoma (9.5%); macular toxoplasmosis (7.1%), and; retinopathy (9.5%) were higher. It is likely that as cataract and uncorrected refractive error become less frequent the relative frequencies of conditions such as age-related macular degeneration and glaucoma will become more prevalent.

It is encouraging to note that only one patient (0.04%) under the age of 18 years had WHO-defined visual impairment, which was due to albinism. There were; however, eleven children (0.44%) who had presenting visual impairment. Each of these cases was due to a refractive error. Table 1; however, demonstrates that the proportion of children under 10 years sampled was lower than the proportion of the children in this age group in the wider population. This may have led to an under-estimation of the level of visual impairment in this age group.

There is a significant effect of age upon visual acuity, whereby the odds of being blind increase by 3% for each year of increased age. (P = 0.002) This is an effect that has been demonstrated previously [2] and alludes to the need for efforts to be focussed on the ageing population, a population for whom it is of paramount importance to achieve their best vision in order to prevent the social and economic costs of visual impairment.

It was demonstrated that females had higher odds of presenting visual impairment than males (OR 1.5, p = 0.02); however there was no effect from general health or ethnic origin. Ethnic origin was not likely to be a factor in the present study due to the widespread mixture of races found in Brazil. However, American studies have shown differences between races where age-related macular degeneration has been more prominent in Caucasians; and cataract and glaucoma have been more significant in African-Americans [11,12].

The effect of work-type on visual impairment found one significant result. It was demonstrated that people who were retired were 2.2 times more likely than professionals to develop visual impairment (P = 0.02). This is likely to reflect the higher prevalence of cataract and retinal disease such as age-related macular degeneration among this population, and again highlights the significant burden that visual impairment will have upon an ageing population.

There are a number of limitations to this study and several methodological issues that could be improved upon in future surveys. Firstly it is possible that individuals with visual problems could have been those more likely to participate in the research. This selection bias could have increased the number of cases and, consequently, overestimated the prevalence of disease in our sample compared to the population from which it was drawn. This is highlighted by the age distribution of the sample. Nearly 50% of the sample was aged over 40 years, whereas in the wider Botucatu population and in the municipality of Sao Paulo approximately 30% of the population was over 40 years (Table 1). More specifically it was also noted that whilst 35.2% of people from the Botucatu census data were aged between 1–19 years, the study sample had only 23.5% of participants in this age category. Similarly 17% of the sampled participants were aged 60 years or over, whereas only 11.2% of the people were in this age category according to census data. The older age of the sample may have lead to an over-estimation of visual impairment and could have artificially inflated the prevalence estimates biasing the observed significant increase in visual impairment with age. These errors in sampling could have been avoided by more accurately collecting the number and age of people within each of the households at the time of visiting the households. This would ensure that only those within the household attended and also afford the opportunity to follow up younger children or adults who may have been at school or work at the time of the examination. A mechanism should have been in place to guarantee that only members of the enumerated households attended for the examination and not neighbours or non-household older relatives who may have been enticed by the offer of ophthalmic assessment.

The methodology of this survey could have been further improved by using the LogMAR visual acuity chart. This has been recommended by the WHO and provides a more accurate assessment of visual acuity and better comparability among studies [13]. Comparability would also have been improved by the employment of a more standardized method to ascribe a primary cause of visual impairment. The causes of visual impairment identified by this study may have been slightly different if WHO definitions and algorithms had been used and glaucoma may have been under-diagnosed without the use of visual field testing. There was also an element of non-random selection introduced by selecting neighboring houses where the actual house occupants refused to participate. It would have been more appropriate to move to the next selected house. This, however; occurred on less than 5% of occasions.

## Conclusion

Blindness according to the WHO criteria was identified in 10 subjects and low vision in 32 subjects. The sampling-design adjusted prevalence of WHO defined blindness was therefore, 0.4% (95% CI: 0.2–0.7) and low vision was 1.3% (95% CI: 0.9–1.7). These results were similar to Iran (0.3% blindness and 1.4% low vision), where the main causes were cataract, AMD, and amblyopia [14]; and comparable to other recently published data where all age groups within a population have been examined. (Table 4) For population-based studies where not all age-groups were included or different definitions of visual impairment were used it is difficult to make comparison with these data. But, for example, in the Blue Mountains Eye Study [15], 0.5% of people over 49 years were blind with best correction compared to 0.8% in this study. Similarly, the Los Angeles Latino Eye Study [16] determined that 0.4% of people over 40 years were blind with best correction compared to 0.5% in Botucatu. Presenting blindness in the Indian State of Andhra Pradesh [17] was found to be 1.84% which is slightly lower than the 2.2% found in this region of Brazil. In Guatemala, Paraguay and Argentina the prevalence of WHO-defined blindness in people 50 years and over was 3.5%, 3.1% and 0.9% respectively [18-20]. In northern Peru, the prevalence of presenting blindness in people 50 years and over was just over 4%, just higher than the 3% found in Botucatu [21].

**Table 4:** Prevalence of presenting blindness in regions where all age groups within the population have been surveyed.

*Year*	*Author*	*Location*	*Sample Size*	*Prevalence of Blindness*
2006	Fotouhi et *al *[14]	Tehran, Iran	4565	0.39%
2006	Schemann et *al *[22]	Cape Verde Islands	3374	0.8%
2007	Shahriari et *al *[23]	Zahedan, Iran	5446	0.79%
	
**Current**	Schellini *et al*	Botucatu, Brazil	2485	2.2%

Presenting blindness is defined as presenting visual acuity < 3/60 in the better eye.

This study demonstrates that non-corrected refractive errors were responsible for the majority of cases of blindness and low vision in this Brazilian population closely followed by cataract. A focus on the optical correction of refractive error and operative intervention in the case of cataract will significantly alleviate the burden of visual impairment in Botucatu, and perhaps wider Brazil. This will be facilitated by greater access to refraction services and a rejuvenation of government funding for the provision of cataract surgery.

## List of abbreviations used

AMD: Age-related macula degeneration; BES: Botucatu Eye Study; CI: Confidence interval; D: Dioptre; IOP: Intraocular pressure; OR: Odds ratio; SD: Standard deviation; SE: Spherical equivalent; VA: Visual acuity; WHO: World health organization.

## Competing interests

The authors declare that they have no competing interests.

## Authors' contributions

SAS made substantial contributions to the conception of the study, acquisition of data, and interpretation of data. SAS was involved in revising the manuscript critically for important intellectual content. SRD made substantial contributions to the analysis and interpretation of data and has been involved in drafting the manuscript and revising it critically for important intellectual content. EH made substantial contributions to conception and design, and acquisition of data. EH has also been involved in drafting the manuscript. FH made substantial contributions to conception and design, acquisition of data, analysis and interpretation of data and has been involved in revising the manuscript critically for important intellectual content. RC made substantial contributions to conception and design, and analysis and interpretation of data, and has been involved in revising the manuscript critically for important intellectual content. RJC has made substantial contributions to analysis and interpretation of data and has been involved in drafting the manuscript and revising it critically for important intellectual content. DS analysis and interpretation of data and has been involved in revising the manuscript critically for important intellectual content. CRP made substantial contributions to conception and design, analysis and interpretation of data and has been involved in revising the manuscript critically for important intellectual content. Each author has participated sufficiently in the work to take public responsibility for appropriate portions of the content. All authors read and approved the final manuscript.

## Pre-publication history

The pre-publication history for this paper can be accessed here:



## Supplementary Material

Additional file 1 **Table S1 – The age-specific prevalence of presenting and WHO-defined low vision, blindness and visual impairment**. The data demonstrate the age-specific prevalence of presenting and WHO-defined low vision, blindness and visual impairment in the surveyed sample. Click here for file

Additional file 2 **Table S2 – Distribution of patients with low vision and blindness according to the disease process diagnosed to be the main cause of visual impairment**. The data in this table demonstrate the distribution of patients with low vision and blindness according to the disease process diagnosed to be the main cause of visual impairment. Click here for file
