# Supplementary material for: Prevalence and causes of visual impairment in a Brazilian population: The Botucatu Eye Study
Source: BMC Ophthalmol. 2009 Aug 19;9:8. doi: 10.1186/1471-2415-9-8 (PMC2734560; doi:10.1186/1471-2415-9-8)
Supplement: Additional file 1 — Table S1 – The age-specific prevalence of presenting and WHO-defined low vision, blindness and visual impairment. The data demonstrate the age-specific prevalence of presenting and WHO-defined low vision, blindness and visual impairment in the surveyed sample. [file 1471-2415-9-8-S1.doc]

**Table 2 - The age-specific prevalence of presenting and WHO-defined low vision, blindness and visual impairment.**

| **Decade of life (years)** | **Presenting**  **Low Vision**  **(%, 95% CI)** | **Presenting Blindness**  **(%, 95% CI)** | **Presenting VI**  **(%, 95% CI)** | **WHO**  **Low Vision**  **(%, 95% CI)** | **WHO Blindness**  **(%, 95% CI)** | **WHO VI**  **(%, 95% CI)** |
| --- | --- | --- | --- | --- | --- | --- |
| All ages  (2485)  Male  Female | 130 (5.2;4.3-6.1)  45 (1.8;13.5-24.2)  85 (3.4;2.8-4.2) | 54 (2.2; 1.6-2.8)  21 (0.9;0.6-1.3)  33 (1.3;0.9-1.9) | 184 (7.4; 6.4-8.5)  66 (2.7;2.1-3.4)  118 (4.7;3.9-5.7) | 32 (1.3;0.9-1.7)  13 (0.5;0.3-0.9)  19 (0.8;0.5-1.2) | 10 (0.4;0.2-0.7)  8 (0.3;0.1-0.6)  2 (0.1;0.0-0.3) | 42 (1.7;1.3-2.3)  21 (0.8;0.6-1.3)  21 (0.8;0.6-1.3) |
| 1-9  (213)  Male  Female | 3 (1.4;0.3-4.3)  2 (0.9;0.0-3.6)  1 (0.5;0.0-2.9) | 1 (0.5;0.0-2.9)  0 (0.0;0.0-1.5)  1 (0.5;0.0-2.9) | 4 (1.9;0.6-4.9)  2 (0.9;0.0-3.6)  2 (0.9;0.0-3.6) | 1 (0.5;0.0-2.9)  1 (0.5;0.0-2.9)  0 (0.0;0.0-1.5) | 0 (0.0;0.0-1.5)  0 (0.0;0.0-1.5)  0 (0.0;0.0-1.5) | 1 (0.5;0.0-2.9)  1 (0.5;0.0-2.9)  0 (0.0;0.0-1.5) |
| 10-19  (372)  Male  Female | 10 (2.7;1.4-4.9)  2 (0.5;0.0-2.1)  8 (2.2;1.0-4.3) | 1 (0.3;0.0-1.7)  0 (0.0;0.0-0.9)  1 (0.3;0.0-1.7) | 11 (2.9;1.6-5.3)  2 (0.5;0.0-2.1)  9 (2.4;1.2-4.6) | 0 (0.0;0.0-0.9)  0 (0.0;0.0-0.9)  0 (0.0;0.0-0.9) | 0 (0.0;0.0-0.9)  0 (0.0;0.0-0.9)  0 (0.0;0.0-0.9) | 0 (0.0;0.0-0.9)  0 (0.0;0.0-0.9)  0 (0.0;0.0-0.9) |
| 20-29  (355)  Male  Female | 18 (5.1;3.2-7.9)  7 (1.9;0.9-4.1)  11 (3.1;1.7-5.5) | 10 (2.8;1.5-5.2)  3 (0.8;0.2-2.6)  7 (1.9;0.9-4.1) | 28 (7.9;5.5-11.2)  10 (2.8;1.5-5.2)  18(5.1;3.2-7.9) | 3 (0.8;0.2-2.6)  1 (0.3;0.0-1.7)  2 (0.6;0.0-2.2) | 2 (0.6;0.0-2.2)  1 (0.3;0.0-1.7)  1 (0.3;0.0-1.7) | 5 (1.4;0.5-3.3)  2 (0.6;0.0-2.2)  3 (0.8;0.2-2.6) |
| 30-39  (317)  Male  Female | 18 (5.7;3.6-8.9)  6 (1.9;0.8-4.2)  12 (3.8;2.1-6.6) | 15 (4.7;2.8-7.7)  4 (1.3;0.4-3.3)  11 (3.4;1.9-6.2) | 33 (10.4;7.5-14.3)  10 (3.2;1.7-5.8)  23 (7.3;4.8-10.7) | 0 (0.0;0.0-1.0)  0 (0.0;0.0-1.0)  0 (0.0;0.0-1.0) | 2 (0.6;0.0-2.4)  2 (0.6;0.0-2.4)  0 (0.0;0.0-1.0) | 2 (0.6;0.0-2.4)  2 (0.6;0.0-2.4)  0 (0.0;0.0-1.0) |
| 40-49  (457)  Male  Female | 22 (4.8;3.2-7.2)  6 (1.3;0.5-2.9)  16 (3.5;2.1-5.7) | 4 (0.9;0.3-2.3)  2 (0.4;0.0-1.7)  2 (0.4;0.0-1.7) | 26 (5.7;3.9-8.2)  8 (1.8;0.8-3.5)  18 (3.9;2.5-6.2) | 1 (0.2;0.0-1.3)  0 (0.0;0.0-0.7)  1 (0.2;0.0-1.3) | 0 (0.0;0.0-0.7)  0 (0.0;0.0-0.7)  0 (0.0;0.0-0.7) | 1 (0.2;0.0-1.3)  0 (0.0;0.0-0.7)  1 (0.2;0.0-1.3) |
| 50-59  (348)  Male  Female | 12 (3.4;1.9-5.9)  4 (1.1;0.3-3.0)  8 (2.3;1.1-4.6) | 4 (1.1;0.3-3.0)  1 (0.3;0.0-1.8)  3 (0.9;0.2-2.6) | 16 (4.6;2.8-7.4)  5 (1.4;0.5-3.4)  11 (3.2;1.7-5.6) | 2 (0.6;0.0-2.2)  2 (0.6;0.0-2.2)  0 (0.0;0.0-0.9) | 0 (0.0;0.0-0.9)  0 (0.0;0.0-0.9)  0 (0.0;0.0-0.9) | 2 (0.6;0.0-2.2)  2 (0.6;0.0-2.2)  0 (0.0;0.0-0.9) |
| 60-69  (216)  Male  Female | 14 (6.5;3.8-10.7)  4 (1.9;0.6-4.8)  10 (4.6;2.4-8.4) | 8 (3.7;1.8-7.3)  4 (1.9;0.6-4.8)  4 (1.9;0.6-4.8) | 22 (10.2;6.8-15.0)  8 (3.7;1.8-7.3)  14 (6.5;3.8-10.7) | 7 (3.2;1.5-6.7)  1 (0.5;0.0-2.8)  6 (2.8;1.1-6.1) | 1 (0.5;0.0-2.8)  1 (0.5;0.0-2.8)  0 (0.0;0.0-1.5) | 8 (3.7;1.8-7.3)  2 (0.9;0.0-3.5)  6 (2.8;1.1-6.1) |
| 70 +  (207)  Male  Female | 33 (15.9;11.5-21.6)  14 (6.8;3.9-11.1)  19 (9.2;5.9-13.9) | 11 (5.3;2.9-9.4)  7 (3.4;1.5-6.9)  4 (1.9;0.6-5.0) | 44 (21.3;16.2-27.4)  21 (10.1;6.7-15.1)  23 (11.1;7.5-16.2) | 18 (8.7;5.5-13.4)  8 (3.9;1.9-7.6)  10 (4.8;2.5-8.8) | 5 (2.4;0.9-5.7)  4 (1.9;0.6-5.0)  1 (0.5;0.0-2.9) | 23 (11.1;7.5-16.2)  12 (5.8;3.3-9.9)  11 (5.3;2.9-9.4) |

CI = confidence interval, VI = visual impairment, WHO = World Health Organization.
