# Supplementary material for: Prevalence and causes of visual impairment in a Brazilian population: The Botucatu Eye Study
Source: BMC Ophthalmol. 2009 Aug 19;9:8. doi: 10.1186/1471-2415-9-8 (PMC2734560; doi:10.1186/1471-2415-9-8)
Supplement: Additional file 2 — Table S2 – Distribution of patients with low vision and blindness according to the disease process diagnosed to be the main cause of visual impairment. The data in this table demonstrate the distribution of patients with low vision and blindness according to the disease process diagnosed to be the main cause of visual impairment. [file 1471-2415-9-8-S2.doc]

**Table 3 -** Distribution of patients with low vision and blindness according to the disease process diagnosed to be the main cause of visual impairment.

| **Eye Pathology** | **Presenting Low Vision**  **(%, 95% CI)** | **Presenting Blindness**  **(%, 95% CI)** | **WHO Low Vision**  **(%, 95% CI)** | **WHO Blindness**  **(%, 95% CI)** | **Presenting VI**  **(%, 95% CI)** | **WHO VI**  **(%, 95% CI)** |
| --- | --- | --- | --- | --- | --- | --- |
| Cataract | 20  (15.5; 10.1-22.7) | 10  (18.5; 10.1-31.0) | 16  (50.0; 33.6-66.4) | 5  (50.0; 23.7-76.3) | 30  (16.3; 11.6-22.4) | 21  (50.0; 35.5-64.5) |
| Refractive Error | 94  (72.3; 64.0-79.3) | 36  (66.7; 53.3-77.8) | 6  (18.8; 8.5-35.7) | 0  (0.0, 0.0-24.9) | 130  (70.7; 63.7-76.8) | 6  (14.4; 6.3-28.2) |
| ARMD | 9  (6.9; 3.5-12.8) | 0  (0.0; 0.0-5.7) | 3  (9.3; 2.5-25.0) | 0  (0.0; 0.0-24.9) | 9  (4.9; 2.5-9.2) | 3  (7.1; 1.8-19.7) |
| Glaucoma | 3  (2.3; 0.5-6.9) | 1  (1.8; 0.0-10.7) | 3  (9.3; 2.5-25.0) | 1  (10.0; 0.0-42.6) | 4  (2.2; 0.7-5.7) | 4  (9.5; 3.2-22.6) |
| Maculopathy | 2  (1.5; 0.1-5.8)[2] | 3  (5.6; 1.3-15.7)[3] | 2  (6.3; 0.7-21.2)[1] | 1  (10.0; 0.0-42.6)[1] | 5  (2.7; 1.0-6.4) | 3  (7.1; 1.8-19.7) |
| Retinopathy | 2  (1.5; 0.1-5.8) | 3  (5.6; 1.3-15.7) | 2  (6.3; 0.7-21.2) | 2  (20.0; 4.6-52.1) | 5  (2.7; 1.0-6.4) | 4  (9.5; 3.2-22.6) |
| Optic Neuropathy | 0  (0.0; 0.0-2.5) | 1  (1.8; 0.0-17.1) | 0  (0.0; 0.0-9.3) | 1  (10.0; 0.0-42.6) | 1  (0.5; 0.0-3.3) | 1  (2.4; 0.0-13.4) |
| **Total** | 130 (100) | 54 (100) | 32 (100) | 10 (100) | 184 (100) | 42 (100) |

Numbers of infectious causes appears in [].CI = Confidence interval, VI = visual impairment, WHO = World Health Organization.
